# Development of risk prediction model for small incision lenticule extraction

**DOI:** 10.3389/fmed.2025.1518889

**Published:** 2025-05-30

**Authors:** Shaowei Zhang, Yulin Yan, Zhengwei Shen, Lei Liu, Pengqi Wang, Jian Zhu, Yanning Yang

**Affiliations:** ^1^Renmin Hospital of Wuhan University, Wuhan, China; ^2^Wuhan Bright Eye Hospital, Wuhan, China; ^3^Tongji Hospital Affiliated to Huazhong University of Science and Technology, Wuhan, China; ^4^Xiangyang Central Hospital, Affiliated Hospital to Hubei University of Arts and Science, Xiangyang, China

**Keywords:** myopia, small-incision lenticule extraction, risk prediction, random forest, retrospective study

## Abstract

**Purpose:**

This study aimed to identify risk factors associated with small-incision lenticule extraction (SMILE) surgery and develop a risk prediction model to aid in determining patient suitability for SMILE.

**Methods:**

This retrospective study included myopia patients from four medical centers in China, enrolled between January 2021 and December 2023. The data were randomly divided into training and test cohorts at an 8:2 ratio. A random forest (RF) model was developed and optimized using three-fold cross-validation, with feature importance assessed.

**Results:**

The study included a total of 2,667 patients, with 2,134 patients in the training cohort and 533 patients in the test cohort. Significant statistical differences were observed in the Belin/Ambrosio Enhanced Ectasia Display and the total deviation value (BAD-D), Corvis Biomechanical Index (CBI), Tomographic and Biomechanical Index (TBI), and spherical equivalent between patients suitable for SMILE and those not suitable, in both the training and test cohorts. The univariate analysis identified ten key features relevant to SMILE. The RF model developed from the training data demonstrated high performance, with an accuracy of 96.0% in the validation set and 95.7% in the test set, an F1 score of 0.967, and an area under the curve (AUC) of 0.976 (95% CI: 0.962–0.990).

**Conclusion:**

SMILE is not appropriate for all patients with myopia. The RF model, based on clinical characteristics, showed excellent performance in predicting SMILE suitability and has potential as a valuable tool for clinical decision-making in the future.

## Background

Myopia is a common refractive disorder in which the eye is too long or the refractive power of the eye’s optical system is too great (usually due to corneal protrusion resulting in excessive corneal curvature), causing images to focus in front of the retina, resulting in blurred vision at a distance (1). Epidemiological studies have shown that the global prevalence of myopia is increasing worldwide (2), and the estimated prevalence would reach 50% in 2050 if no effective intervention was performed (3). In the United States, the prevalence of myopia in people aged 12 to 54 years nearly doubled from 1971–1972 to 1999–2004 (25 to 42%) (4). The prevalence of myopia also varies by race and ethnicity. Chiang et al. (5) showed that the prevalence of myopia in other races, Hispanic, Mexican American, non-Hispanic Black, and non-Hispanic White were 42.77, 38.43, 34.23, 32.26, and 31.06%, respectively. And the worldwide prevalence of myopia is particularly high in Asians (6). Treatment of myopia depends on individuals’ symptoms and needs (7). The overall goals of treatment are to improve visual acuity, visual comfort (e.g., visual distortion, polymyalgia, decreased stereopsis), and other visual functions (e.g., color discrimination, motion detection, peripheral vision). First-line treatment includes corrective lenses, such as spectacles and contact lenses, or refractive surgery (8).

Small incision intrastromal lenticule extraction (SMILE) is one kind of refractive surgery performed using a femtosecond laser, and its early results were first reported in 2011 (9). SMILE became available in 2012, and the U.S. Food and Drug Administration (FDA) approved its use in the United States in September 2016. In a review of outcomes of SMILE patients, Song concluded that SMILE provided effective and predictable results and generally had equivalent outcomes with another refractive surgery option named laser-assisted stromal *in situ* keratomileusis (LASIK) (10). The long-term refractive regression was 0.48 diopters 5 years after surgery compared with 6 months after surgery, and there was no decrease in corrected distance visual acuity by two or more lines over the 5 years (11). Therefore, SMILE is now considered a safe and effective alternative to LASIK and photorefractive keratectomy (PRK) (12).

There are systemic and ocular contraindications to refractive surgery. Autoimmune diseases, collagen vascular diseases, and immunodeficiency disorders can impair corneal healing (13). Pregnant or lactating women can experience fluctuations in vision due to changes in corneal hydration causing refractive changes in the eye (14). Patients with scar tissue who have abnormal wound healing (e.g., keloids or abnormal scar formation) may experience abnormal corneal healing (15). Systemic medications such as oral isotretinoin can exacerbate dry eye symptoms, and amiodarone can leave transient corneal epithelial deposits (16). Although the U.S. FDA labeling contains a warning against laser refractive surgery in patients with diabetes, a literature review found that LASIK and PRK can be performed without complications in patients with well-controlled diabetes who do not have cataracts, diabetic retinopathy, or systemic complications of diabetes (17).

In summary, preoperative evaluation is crucial for decision-making in refractive surgery. This study focused on myopic patients seeking SMILE surgery to identify key factors influencing surgical suitability based on initial clinical examinations and medical history. By developing a machine learning model, we aimed to assist clinicians in determining the appropriateness of SMILE surgery for individual patients.

## Methods

### Study design and participants

This study included myopic patients who visited to Xiangyang Central Hospital, Affiliated Hospital to Hubei University of Arts and Science, Renmin Hospital of Wuhan University, Wuhan Bright eye hospital, and Tongji Hospital affiliated with Tongji Medical College of Huazhong University of Science and Technology from January 2021 to December 2023. The inclusion criteria were: (1) Myopic patients who intend to receive SMILE surgery; (2) Aged over 18 years or older; (3) Patients with relatively stable diopters, with recommended parameters: spherical equivalent within −0.75 to −10D, simple sphere from −0.5 to −10D, and astigmatism less than 5D. The exclusion criteria were: (1) Patients using systemic glucocorticoids or immunosuppressants, etc.; (2) Patients with diabetes, history of tumors, autoimmune diseases (including systemic lupus erythematosus, rheumatoid arthritis, multiple sclerosis, Hashimoto’s thyroid disease), scar constitution, mental illness; (3) Pregnant or lactating women. This study was approved by the Ethics Committee of Xiangyang Central Hospital, and informed consent was waived due to the retrospective nature of the study.

### Data collection and definitions

Baseline data were obtained from the medical history system and included the following variables: age, gender, occupation, and history of local eye diseases. Ophthalmological examination data collected comprised uncorrected visual acuity (UCVA), best corrected distance visual acuity (BCDVA), astigmatism, intraocular pressure (IOP), spherical, cylinder, axis, funduscopy evaluation, corneal thickness and diameter, corneal ectasia or other corneal degeneration, dry eye evaluation, slit lamp examination, dark pupil diameter (refers to the pupil diameter of patients in dark rooms), corneal topography data, and the tendency of keratoconus.

Patients were categorized into two groups: those suitable for SMILE surgery and those not suitable. Experienced ophthalmologists made a comprehensive assessment based on ocular examinations, which included corneal topography and biomechanics, degree of myopia, and corneal thickness.

### Model development and validation

The entire cohort was randomly divided into a training set and a test set at an 8:2 ratio. A Random Forest (RF) model was utilized, with hyperparameter optimization performed using three-fold cross-validation. The final model was trained on the entire training cohort with the optimized parameters, and feature importance was assessed. The model’s performance was evaluated on the test set, with predictions compared against actual outcomes. The Receiver Operating Characteristic (ROC) curve was plotted, and the Area Under the Curve (AUC) was used as the primary evaluation metric.

### Statistical analysis

All statistical analyses in this study were performed using R software (version 4.2.3). Continuous variables were expressed as mean ± SD or median (IQR). Categorical variables were expressed as frequency and percentage *n* (%). Continuous variables were first tested for normality. If they were normally distributed, the *t-*test was used for comparison between the two groups. If they were not normally distributed, the Wilcoxon-Mann-Whitney test was used for comparison between the two groups. Categorical variables were analyzed using the chi-square test or Fisher’s exact value method. Factors with a miss rate > 20% were directly removed for further analysis, for data with a miss rate < 20%, the multiple imputation by chained equations (MICE) algorithm was conducted with the following parameters: five imputed datasets (*m* = 5) were generated through five iterations (maxit = 5) using the predictive mean matching (PMM) method. Following imputation, the convergence and consistency across the imputed datasets was assessed and one complete dataset with optimal stability was selected for subsequent analyses. The univariate analysis was used to explore the risk factors for the SMILE surgery. A logistic regression model was further built on the entire training set using the glm method and selected factors. Following that, bidirectional stepwise regression was performed based on the initial model, with the optimal bidirectional stepwise regression model selected as the final model according to the Akaike Information Criterion (AIC). The trained model was applied to the test set, the F1-score and area under the curve (AUC) were used for the evaluation of the developed prediction model. In this study, a *p* < 0.05 indicated a statistical significance.

## Results

### Patients’ characteristics

A total of 2,667 patients were included in the analysis. The results revealed that patients not suitable for SMILE surgery had higher uncorrected visual acuity (UCVA) (0.06 vs. 0.04, *p* < 0.001), higher intraocular pressure (IOP) (18.00 vs. 17.25, *p* < 0.001), and a higher spherical equivalent (−5.28 vs. −4.75, *p* < 0.001), as well as lower K1, K2, and Km values. In terms of corneal characteristics, the non-SMILE group exhibited a larger radius of curvature, higher BAD-D (2.38 vs. 1.17, *p* < 0.001), higher CBI (0.62 vs. 0.02, *p* < 0.001), higher TBI (0.86 vs. 0.13, *p* < 0.001), and a larger dark pupil diameter (6.59 vs. 6.37, *p* < 0.001) (Table 1).

**Table 1:** Clinical characteristics for all participants.

Variables	Not suitable for SMILE (*n* = 975)	Suitable for SMIL (*n* = 1,692)	*p*-value
UCVA	0.06 (0.04, 0.10)	0.04 (0.02, 0.10)	<0.001
BSCVA	1.00 (1.00, 1.00)	1.00 (1.00, 1.00)	<0.001
IOP	18.00 (15.85, 20.00)	17.25 (15.00, 19.30)	<0.001
Spherical	-4.75 (−7.50, −3.50)	−4.50 (−5.50, −3.50)	<0.001
Cylinder	−0.50 (−0.75, 0.00)	−0.50 (−1.00, −0.25)	0.013
Axis	75.00 (0.00, 170.00)	80.00 (5.00, 170.00)	0.122
Spherical equivalent	−5.38 (−8.50, −4.00)	−4.75 (−5.88, −3.75)	<0.001
K1	42.70 (41.60, 44.10)	43.10 (42.20, 43.80)	<0.001
Axial K1	98.90 (8.00, 172.00)	98.80 (8.00, 171.70)	0.950
K2	43.90 (42.70, 45.20)	44.20 (43.20, 45.00)	0.006
Axial K2	89.70 (81.95, 97.65)	89.80 (81.50, 97.90)	0.855
Km	43.25 (42.17, 44.62)	43.60 (42.65, 44.40)	0.002
Mean radius of curvature	7.81 (7.57, 8.01)	7.74 (7.60, 7.91)	0.002
WTW	11.60 (11.30, 11.90)	11.60 (11.40, 11.90)	0.004
Preoperative Corneal thickness, μm	538.00 (508.00, 559.00)	547.00 (528.00, 564.00)	<0.001
BAD-D	2.38 (1.40, 2.97)	1.17 (0.96, 1.42)	<0.001
CBI	0.62 (0.23, 0.82)	0.02 (0.00, 0.13)	<0.001
TBI	0.86 (0.36, 1.00)	0.13 (0.04, 0.25)	<0.001
Dark pupil diameter, mm	6.59 (6.12, 7.16)	6.37 (5.95, 6.73)	<0.001
Schirmer’s tear test, mm	14.00 (12.00, 16.00)	14.00 (12.00, 15.00)	0.201
Dominant eye, *n* (%)			0.145
Left	745 (76.41)	1,334 (78.84)	
Right	230 (23.59)	358 (21.16)	
Astigmatism, *n* (%)	783 (80.31)	1,382 (81.68)	0.383

BAD-D, Belin/Ambrosio Enhanced Ectasia Display and the total deviation value; CBI, Corvis Biomechanical Index; IOP, Intraocular pressure; TBI, Tomographic and Biomechanical Index; UCVA, uncorrected visual acuity, BSCVA, best spectacle-corrected visual acuity.

After splitting the data, the training cohort comprised 2,134 patients, while the test cohort had 533 patients. Despite the random split, the characteristics of patients in the training and test cohorts were similar to those of the overall cohort. Significant differences remained for BAD-D (training: 1.17 vs. 2.37; test: 1.19 vs. 2.48, both *p* < 0.001), CBI (training: 0.02 vs. 0.61; test: 0.04 vs. 0.66, both *p* < 0.001), TBI (training: 0.13 vs. 0.86; test: 0.14 vs. 0.88, both *p* < 0.001), and spherical equivalent (training: −5.50 vs. −4.75; test: −5.12 vs. −5.00, both *p* < 0.001). Detailed information is provided in Tables 2, 3.

**Table 2:** Clinical characteristics for participants in the training cohort.

Variables	Not suitable for SMILE (*n* = 780)	Suitable for SMILE (*n* = 1,354)	*p*-value
UCVA	0.06 (0.03, 0.10)	0.04 (0.02, 0.10)	0.001
BSCVA	1.00 (1.00, 1.00)	1.00 (1.00, 1.00)	<0.001
IOP	18.00 (15.88, 20.00)	17.40 (15.20, 19.40)	0.001
S	−4.75 (−7.75, −3.50)	−4.50 (−5.50, −3.50)	<0.001
C	−0.50 (−0.75, 0.00)	−0.50 (−1.00, −0.25)	0.052
A	72.50 (0.00, 170.00)	80.00 (5.00, 170.00)	0.277
Spherical equivalent	−5.50 (−8.50, −4.00)	−4.75 (−5.88, −3.75)	<0.001
K1	42.70 (41.60, 44.20)	43.10 (42.20, 43.88)	0.006
Axial K1	111.55 (7.77, 172.62)	92.80 (8.30, 171.57)	0.579
K2	43.90 (42.70, 45.20)	44.20 (43.20, 45.00)	0.027
Axial K2	89.30 (82.00, 97.23)	89.90 (81.53, 98.30)	0.352
Km	43.30 (42.14, 44.65)	43.60 (42.65, 44.45)	0.012
Mean radius of curvature	7.81 (7.59, 8.02)	7.74 (7.61, 7.91)	0.001
WTW	11.60 (11.30, 11.90)	11.60 (11.40, 11.90)	0.003
Preoperative Corneal thickness, μm	538.00 (508.00, 559.00)	547.00 (529.00, 564.00)	<0.001
BAD-D	2.37 (1.39, 2.97)	1.17 (0.94, 1.42)	<0.001
CBI	0.61 (0.21, 0.81)	0.02 (0.00, 0.12)	<0.001
TBI	0.86 (0.35, 1.00)	0.13 (0.04, 0.25)	<0.001
dark pupil diameter, mm	6.61 (6.13, 7.18)	6.36 (5.93, 6.73)	<0.001
Schirmer’s tear test, mm	14.00 (12.00, 15.00)	14.00 (12.00, 15.75)	0.089
Dominant eye, *n* (%)			0.389
Left	602 (77.2)	1,068 (78.9)	
Right	178 (22.8)	286 (21.1)	
Astigmatism, *n* (%)	626 (80.36)	1,106 (81.68)	0.428

BAD-D, Belin/Ambrosio Enhanced Ectasia Display and the total deviation value; CBI, Corvis Biomechanical Index; IOP, Intraocular pressure; TBI, Tomographic and Biomechanical Index; UCVA, uncorrected visual acuity, BSCVA, best spectacle-corrected visual acuity.

**Table 3:** Clinical characteristics for participants in the test cohort.

Variables	Not suitable for SMILE (*n* = 195)	Suitable for SMILE (*n* = 338)	*p*-value
UCVA	0.04 (0.04, 0.11)	0.04 (0.02, 0.10)	0.129
BSCVA	1.00 (1.00, 1.00)	1.00 (1.00, 1.00)	0.037
IOP	18.00 (15.90, 19.00)	17.00 (15.00, 19.08)	0.195
S	−4.75 (−6.88, −3.50)	−4.75 (−5.50, −3.50)	0.056
C	−0.50 (−0.75, 0.00)	−0.50 (−1.00, −0.25)	0.098
A	75.00 (0.00, 167.50)	90.00 (5.50, 170.00)	0.179
Spherical equivalent	−5.12 (−7.62, −4.00)	−5.00 (−6.00, −3.75)	<0.001
K1	42.70 (41.75, 43.80)	43.20 (42.30, 43.80)	0.014
Axial K1	59.50 (8.55, 168.45)	112.40 (7.62, 172.40)	0.223
K2	43.80 (42.80, 44.90)	44.20 (43.30, 44.90)	0.067
Axial K2	91.80 (81.45, 98.90)	89.50 (81.50, 96.80)	0.141
Km	43.10 (42.38, 44.23)	43.65 (42.71, 44.30)	0.037
Mean radius of curvature	7.80 (7.52, 8.00)	7.71 (7.59, 7.88)	0.293
WTW	11.60 (11.30, 11.90)	11.60 (11.40, 11.80)	0.528
Preoperative Corneal thickness, μm	537.00 (506.00, 562.00)	544.00 (527.00, 560.75)	0.001
BAD-D	2.48 (1.48, 2.98)	1.19 (0.98, 1.39)	<0.001
CBI	0.66 (0.25, 0.86)	0.04 (0.00, 0.15)	<0.001
TBI	0.88 (0.45, 1.00)	0.14 (0.04, 0.25)	<0.001
dark pupil diameter, mm	6.53 (5.96, 7.04)	6.39 (6.00, 6.81)	0.016
Schirmer’s tear test, mm	14.00 (12.00, 17.00)	14.00 (12.00, 15.00)	0.596
Dominant eye, *n* (%)			0.192
Left	143 (73.3)	266 (78.7)	
Right	52 (26.7)	72 (21.3)	
Astigmatism, *n* (%)	157 (80.51)	276 (81.66)	0.657

BAD-D, Belin/Ambrosio Enhanced Ectasia Display and the total deviation value; CBI, Corvis Biomechanical Index; IOP, Intraocular pressure; TBI, Tomographic and Biomechanical Index; UCVA, uncorrected visual acuity, BSCVA, best spectacle-corrected visual acuity.

### Univariate analysis for SMILE surgery

The univariate analysis identified ten factors associated with suitability for SMILE surgery, including BSCVA (OR: 18.96, 95%CI: 9.48 ~ 37.93, *p* < 0.001), IOP (OR: 0.95, 95%CI: 0.93 ~ 0.97, *p* < 0.001), S (OR: 1.15, 95%CI: 1.12 ~ 1.19, *p* < 0.001), spherical equivalent (OR: 1.15, 95%CI: 1.22 ~ 1.31, *p* < 0.001), WTW (OR:1.37, 95%CI: 1.12 ~ 1.68, *p* = 0.002), preoperative corneal thickness (OR:1.02, 95%CI: 1.01 ~ 1.02, *p* < 0.001), BAD-D (OR: 0.11, 95%CI: 0.09 ~ 0.13, *p* < 0.001), CBI (OR: 0.00, 95%CI: 0.00 ~ 0.00, *p* < 0.001), TBI (OR: 0.00, 95%CI: 0.00 ~ 0.00, *p* < 0.001), dark pupil diameter (OR: 0.57, 95%CI: 0.50 ~ 0.64, *p* < 0.001), and Schirmer’s tear test (OR: 1.04, 95%CI: 1.02 ~ 1.06, *p* < 0.001) (Table 4).

**Table 4:** Univariate analysis for surgery decision.

Variables	*p*-value	OR (95%CI)
UCVA	0.236	0.62 (0.28 ~ 1.37)
BSCVA	<0.001	18.96 (9.48 ~ 37.93)
IOP	<0.001	0.95 (0.93 ~ 0.97)
S	<0.001	1.15 (1.12 ~ 1.19)
C	0.094	0.89 (0.77 ~ 1.02)
A	0.245	1.00 (1.00 ~ 1.00)
Spherical equivalent	<0.001	1.27 (1.22 ~ 1.31)
K1	0.439	1.02 (0.97 ~ 1.07)
Axial K1	0.923	1.00 (1.00 ~ 1.00)
K2	0.540	0.99 (0.94 ~ 1.03)
Axial K2	0.369	1.00 (1.00 ~ 1.00)
Km	0.977	1.00 (0.95 ~ 1.05)
The mean radius of curvature	0.516	0.91 (0.68 ~ 1.21)
WTW	0.002	1.37 (1.12 ~ 1.68)
Preoperative Corneal thickness, μm	<0.001	1.02 (1.01 ~ 1.02)
BAD-D	<0.001	0.11 (0.09 ~ 0.13)
CBI	<0.001	0.00 (0.00 ~ 0.00)
TBI	<0.001	0.00 (0.00 ~ 0.00)
Dark pupil diameter, mm	<0.001	0.57 (0.50 ~ 0.64)
Schirmer’s tear test, mm	<0.001	1.04 (1.02 ~ 1.06)
Dominant eye (right)	0.145	0.87 (0.72 ~ 1.05)
Presence of astigmatism	0.383	1.09 (0.89 ~ 1.34)

BAD-D, Belin/Ambrosio Enhanced Ectasia Display and the total deviation value; CBI, Corvis Biomechanical Index; IOP, Intraocular pressure; TBI, Tomographic and Biomechanical Index; UCVA, uncorrected visual acuity, BSCVA, best spectacle-corrected visual acuity.

### Model performance

**Figure 1:**
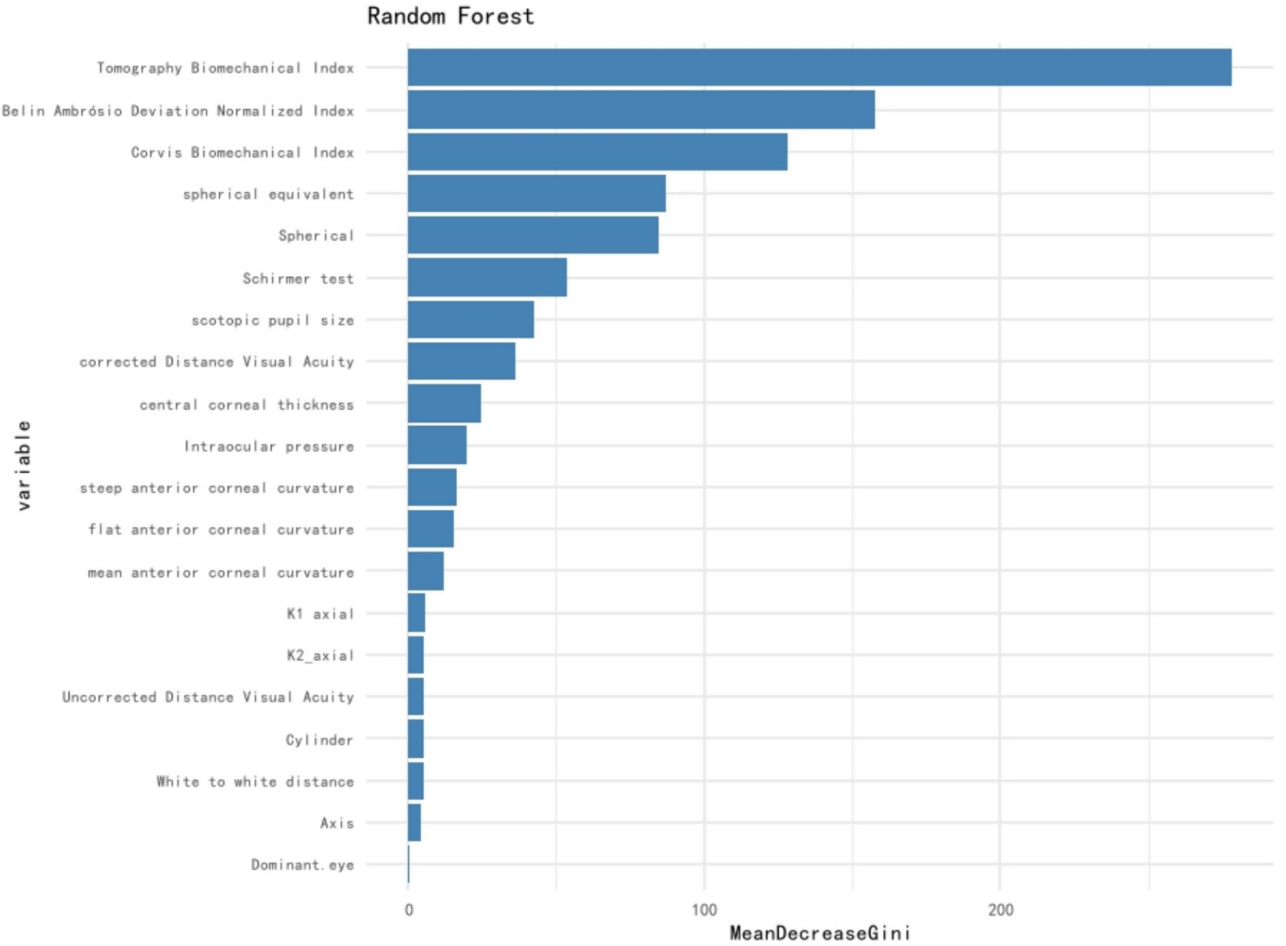
Feature importance for the random forest model.

**Figure 2:**
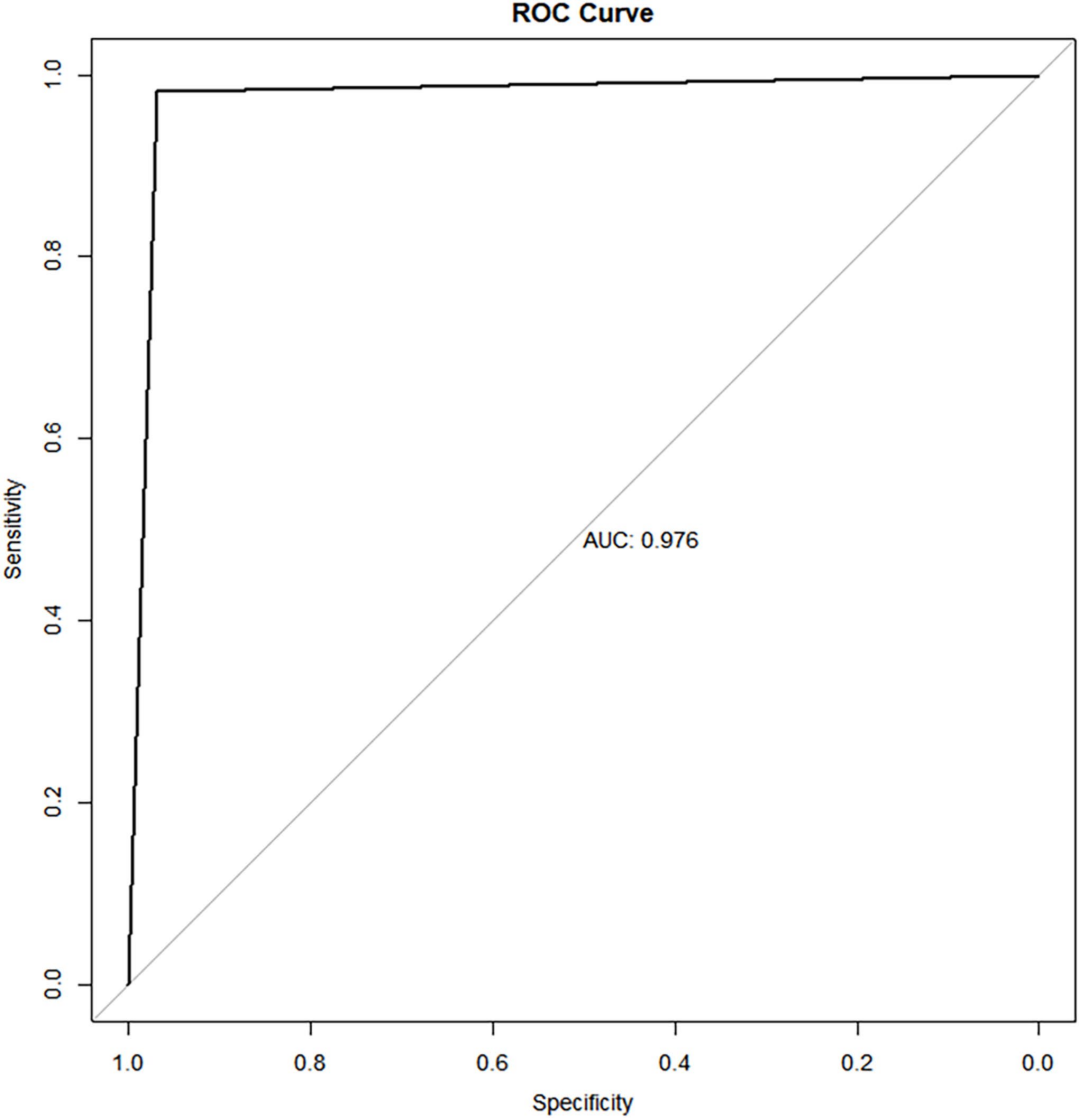
ROC curve of the random forest model for surgery decision prediction, the AUC was 0.976 (95%CI:0.962–0.990).

**Figure 3:**
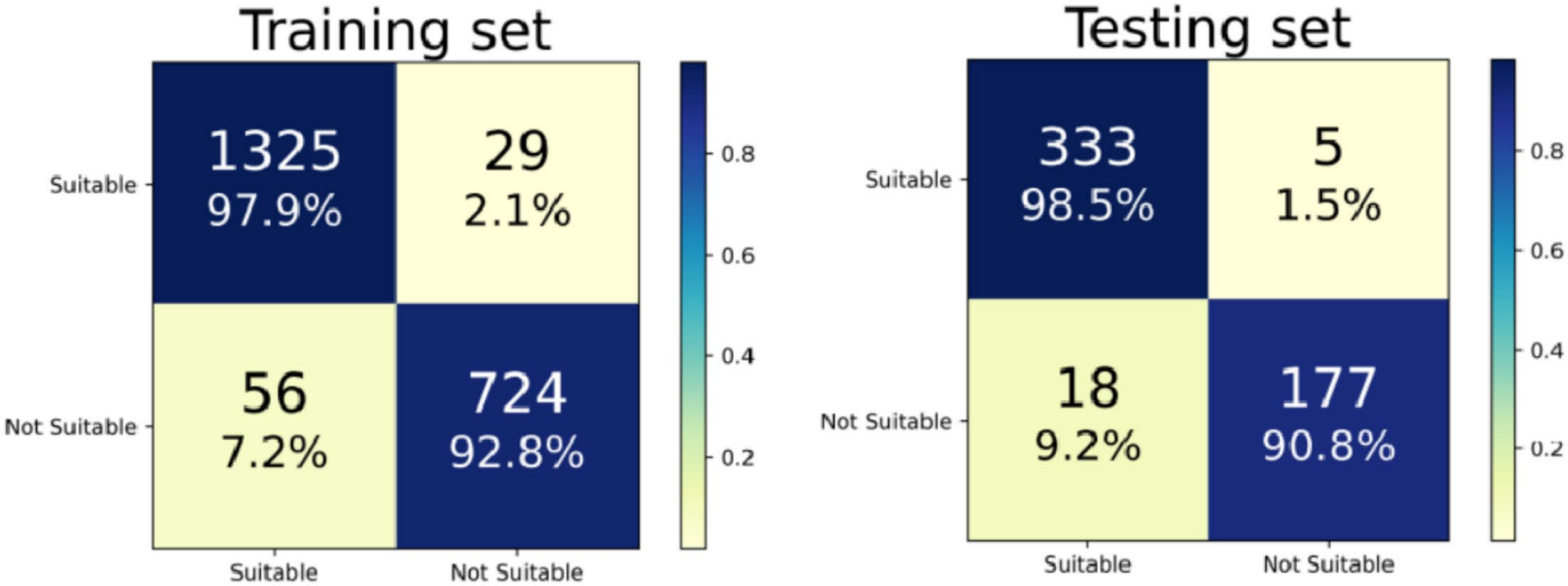
Confusion matrices of the training set and testing set.

An RF model was developed and fine-tuned. The analysis identified the top five features that most significantly reduced the Gini coefficient: TBI, BAD-D, CBI, spherical equivalent, and spherical (Figure 1). This finding supports our previous analysis of the differing characteristics between the non-SMILE and SMILE groups. Cross-validation results indicated an accuracy of 96.0%, while the test results showed an accuracy of 95.7%. The F1 score was 0.967, and the AUC was 0.976 (Figure 2). The confusion matrix is provided in Figure 3.

**Figure 4:**
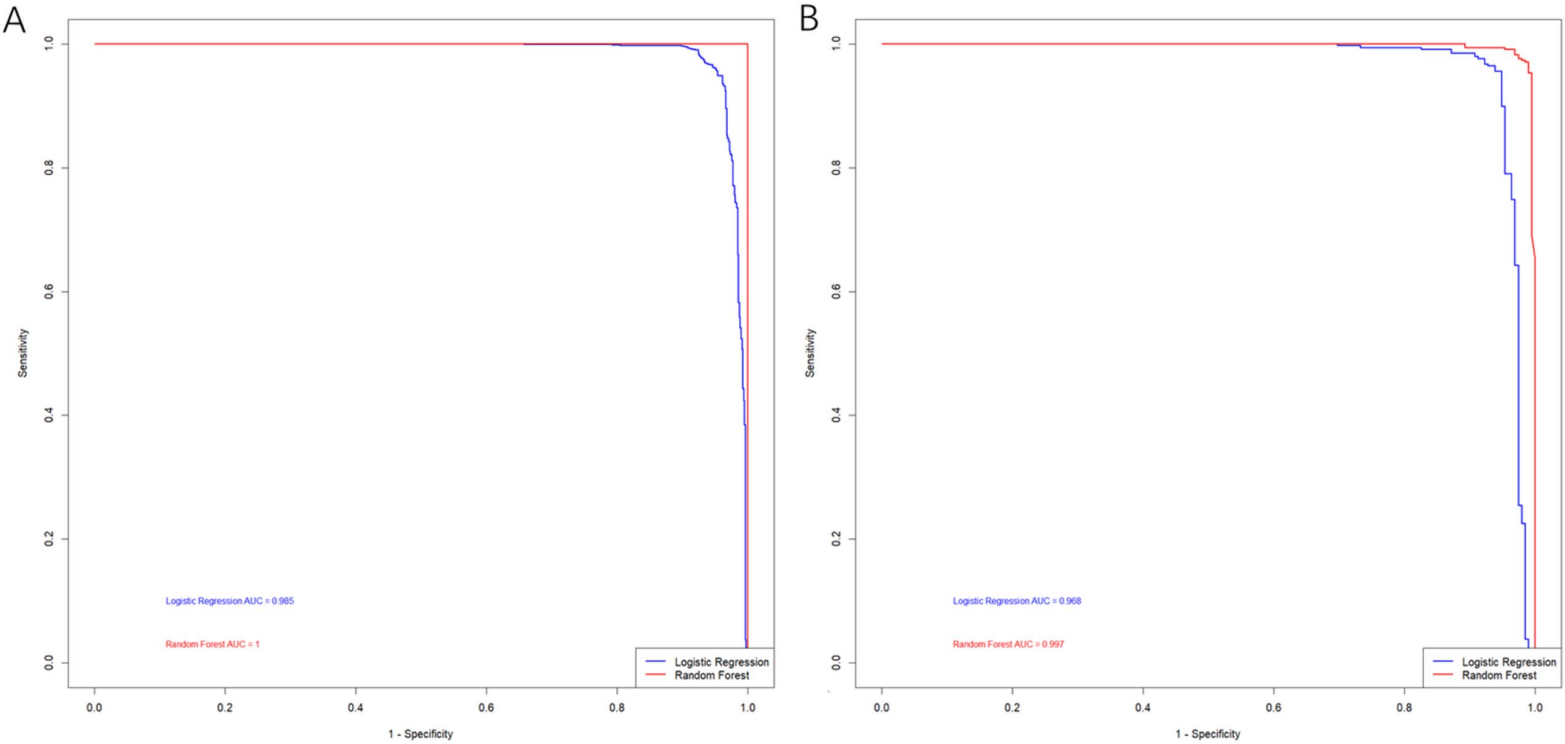
ROC curve of the random forest model and logistic regression model. **(A)** Training set; **(B)** Testing set.

The comparison of the two models showed that the RF model outperformed the logistic regression model in both the training and testing datasets, with AUC values of 1.000 versus 0.985 for the training set and 0.997 versus 0.968 for the testing set (Figure 4).

## Discussion

This study identified BSCVA, IOP, S, spherical equivalent, preoperative corneal thickness, BAD-D, CBI, TBI, dark pupil diameter, and Schirmer’s tear test as important factors for determining SMILE surgery suitability. Using these factors, a RF model was developed for predicting non-SMILE cases. The model achieved an AUC of 0.976, with an accuracy of 97.4% and an F1 score of 97.7%. These results suggest that machine learning methods could be a valuable tool for future surgical decision-making.

SMILE is an “all-in-one” surgical method for refractive correction that has been widely adopted worldwide. However, several risk factors have been associated with SMILE surgery and its prognosis. Lee et al. (18) found that steep corneal curvature is linked to a higher risk of significant postoperative residual astigmatism after SMILE surgery, Yang et al. (19) reported that steep corneal curvature, thin cap thickness, and high preoperative spherical equivalent are potential risk factors for the formation of an opaque bubble layer during SMILE surgery. Zhao et al. (20) demonstrated that the spherical equivalent is correlated with surgical complications. Consistent with these studies, our results indicated that the spherical equivalent differed significantly between patients suitable for SMILE and those not suitable, even after data splitting, with a *p* value < 0.001. Furthermore, the spherical equivalent was identified as a risk factor for SMILE surgery, with an OR of 1.27 (95% CI: 1.22–1.31, *p* < 0.001).

CBI, TBI, and BAD-D are biomechanical features widely used for evaluating myopia, SMILE surgery, and other ocular diseases. Zarei-Ghanavati et al. (21) conducted a study where participants received a 110-μm cap thickness in one eye and a 145-μm cap thickness in the fellow eye. Three months post-surgery, researchers found a significant difference in the CBI (21). Vinviguerra et al. (22) suggested that CBI-related techniques could be useful for managing patients who underwent keratectomy. Corral et al. explored the changes in CBI and TBI in patients who successfully underwent orthokeratology (23). Additionally, Zhang et al. (24) used BAD-D, CBI, and TBI in combination as early predictive indicators for myopia patients with keratoconus before refractive surgery, showing that TBI, CBI, and BAD-D had excellent diagnostic efficiency. In our study, aside from the spherical equivalent, CBI, TBI, and BAD-D were consistently significant (*p* < 0.001) in both the training and test cohorts. The univariate analysis for these three factors also indicated their potential as useful features for SMILE surgery decision-making.

RF is a well-established machine learning algorithm with extensive applications across medical science. From the perspectives of myopia and SMILE surgery, several studies have demonstrated its efficacy. Xu et al. (25) employed the RF model to explore changes in pupil diameter among myopic preschoolers. Wang et al. (26) developed various machine learning models and found that the RF model performed best in estimating lenticule thickness in SMILE surgery. Additionally, Li et al. (27) created an RF-based model for selecting refractive surgery based on clinical data, which showed a strong agreement with ophthalmologists, with a performance metric of 0.8775. Given that RF is a validated and robust model across diverse datasets, the current study did not compare RF with other models and we will incorporate other machine learning models in future analysis. Nonetheless, the RF model demonstrated excellent performance in terms of both AUC and accuracy in this study. The development of this prediction model had great clinical potential, for patients who consider the SMILE operation, the model could provide a personalized prediction, although the prognostic of SMILE was not included in the present study, the model correlation well with the opinion of experienced ophthalmologists, which was of ultimate importance for medical centers with limited medical resources.

Except for the features selected, there were many other reasons that could affect the execution of SMILE surgery, including patients’ willingness, financial status, and occupation requirement; all these factors were not included for the analysis; therefore, more detailed inclusion and exclusion criteria should be made for future studies. Moreover, to exclude potential confounding factors, mediation analysis should also be considered in future analyses. Consejo et al. (28) found that age is significantly correlated with both corneal tilt and corneal densitometry, and the interaction between corneal tilt and corneal densitometry is strongly influenced by age, in the present study, many parameters were included, and the mediation analysis could help filter features with greater importance.

However, this study has several limitations. Firstly, despite including patients from multiple medical centers, the retrospective nature of the study introduces selection bias, also, to improve the model performance, we did not select some data or further include data from other medical centers as an independent external validation set, which could limit the generalization ability of the present study. Secondly, the determination of suitability for SMILE surgery was made by experienced ophthalmologists, making the results heavily reliant on their clinical judgment and expertise. Thirdly, due to time constraints, we were unable to perform a prognosis-related analysis. Investigating the risk factors associated with SMILE surgery outcomes would be highly valuable for future research.

## Conclusion

This study showed that factors such as BSCVA, IOP, S, spherical equivalent, preoperative corneal thickness, BAD-D, CBI, TBI, dark pupil diameter, and the Schirmer’s tear test are crucial in determining suitability for SMILE surgery. Additionally, the RF model was found to be an effective tool for aiding surgical decision-making. However, the retrospective design of this study limits its clinical applicability, highlighting the need for a larger prospective study to validate these findings.

## Data Availability

The original contributions presented in the study are included in the article/supplementary material, further inquiries can be directed to the corresponding authors.
